# Editing of *StSR4* by Cas9-RNPs confers resistance to *Phytophthora infestans* in potato

**DOI:** 10.3389/fpls.2022.997888

**Published:** 2022-09-23

**Authors:** Ki-Beom Moon, Su-Jin Park, Ji-Sun Park, Hyo-Jun Lee, Seung Young Shin, Soo Min Lee, Gyung Ja Choi, Sang-Gyu Kim, Hye Sun Cho, Jae-Heung Jeon, Yong-Sam Kim, Youn-Il Park, Hyun-Soon Kim

**Affiliations:** ^1^ Plant Systems Engineering Research Center, Korea Research Institute of Bioscience and Biotechnology, Daejeon, Republic of Korea; ^2^ Department of Biosystems and Bioengineering, KRIBB School of Biotechnology, University of Science and Technology, Daejeon, Republic of Korea; ^3^ Department of Functional Genomics, KRIBB School of Bioscience, University of Science and Technology, Daejeon, Republic of Korea; ^4^ Center for Eco-Friendly New Materials, Korea Research Institute of Chemical Technology, Daejeon, Republic of Korea; ^5^ Department of Biological Sciences, Korea Advanced Institute for Science and Technology, Daejeon, Republic of Korea; ^6^ Genome Editing Research Center, Korea Research Institute of Bioscience and Biotechnology, Daejeon, Republic of Korea; ^7^ GenKOre, Daejeon, Republic of Korea; ^8^ Department of Biological Sciences, Chungnam National University, Daejeon, Republic of Korea

**Keywords:** RNPs, protoplast, genome editing, susceptibility gene, late blight

## Abstract

Potato (*Solanum tuberosum* L.) cultivation is threatened by various environmental stresses, especially disease. Genome editing technologies are effective tools for generating pathogen-resistant potatoes. Here, we established an efficient RNP-mediated CRISPR/Cas9 genome editing protocol in potato to develop *Phytophthora infestans* resistant mutants by targeting the susceptibility gene, *Signal Responsive 4* (*SR4*), in protoplasts. Mutations in *StSR4* were efficiently introduced into the regenerated potato plants, with a maximum efficiency of 34%. High co-expression of *StEDS1* and *StPAD4* in *stsr4* mutants induced the accumulation of salicylic acid (SA), and enhanced the expression of the pathogen resistance marker *StPR1*. In addition, increased SA content in the *stsr4* mutant enhanced its resistance to *P. infestans* more than that in wild type. However, the growth of *stsr4_3-19* and *stsr4_3-698* mutants with significantly high SA was strongly inhibited, and a dwarf phenotype was induced. Therefore, it is important to adequate SA accumulation in order to overcome *StSR4* editing-triggered growth inhibition and take full advantages of the improved pathogen resistance of *stsr4* mutants. This RNP-mediated CRISPR/Cas9-based potato genome editing protocol will accelerate the development of pathogen-resistant *Solanaceae* crops *via* molecular breeding.

## Introduction

Plants evolve innate immune systems to defend against pathogens. However, most plant pathogens that escape or suppress plant immunity establish a supply system in the host plant in cooperation with plant cells to derive nutrition, thus ensuring their survival and maintenance. The host plant genes that promote pathogen infection and aid in its survival and maintenance are called susceptibility (*S*) genes (van Schie and Takken, 2014). Loss of susceptibility function by *S* gene modification could be a practical and alternative approach for improving the pathogen tolerance of the plant because *S* genes no longer facilitate infection and support compatibility of the pathogen (Jørgensen, 1992; Vogel et al., 2002; van Schie and Takken, 2014). Many studies use genome editing tools for developing pathogen-resistant plants by targeting the *S* genes. For example, *MLO* and *eIF4* confer resistance to powdery mildew (Malnoy et al., 2016; Nekrasov et al., 2017) and potyvirus (Pyott et al., 2016), respectively. Furthermore, *SWEET* and *DMR6* confer resistance to *Xanthomonas* bacteria (Oliva et al., 2019) and a broad spectrum of bacterial and fungal pathogens (de Toledo Thomazella et al., 2021), respectively. In particular, *DND1*, *CHL1*, and *DMR6* are known as *S* genes that confer resistance to *P. infestans* in potato (Kieu et al., 2021).

The Signal Response 1 (SR1) protein, also known as calmodulin (CaM)-binding transcription activator 3 (CAMTA3), mediates the inhibition of effector-triggered immunity, and plays an essential role in hypersensitive response signaling by binding to the promoters of *enhanced disease susceptibility 1* (*EDS1*) and *nonrace-specific disease resistance 1* (*NDR1*) (Du et al., 2009; Nie et al., 2012). Mutated *Arabidopsis thaliana* (*Atsr1*) exhibit enhanced disease resistance against several pathogens, such as *Pseudomonas syringae* pv. *tomato* DC3000, *Golovinomyces cichoracearum*, and *Botrytis cinerea* (Galon et al., 2008; Du et al., 2009; Nie et al., 2012) by elevating salicylic acid (SA) levels. However, side effects caused by mutations in *S* genes must be considered because *S* gene-mediated resistance is often accompanied by a decrease in growth, yield, and fertility (Zaidi et al., 2018). Phylogenetic analysis of data available from the potato genome database reveals that *StSR4* (PGSC0003DMP400026345, Sotub01g012330) is the CAMTA3 gene, and the deduced amino acid sequence of *StSR4* is most similar to that of *A. thaliana* SR1 (Sun et al., 2016). Sun et al. (2016) report that RNAi silencing of *StSR4* enhances late blight resistance in potato. Although *StSR4*-silenced plants enhanced resistance to the *P. infestans*, they are designated as genetically-modified organisms (GMOs). Therefore, regulatory restrictions for GMOs limit their use in commercial agricultural settings.

Late blight is caused by the pathogen *P. infestans*. It is one of the most devastating bacterial diseases worldwide, resulting in yield losses of 40% in susceptible potato cultivars (Arévalo-Marín et al., 2021). One of the main strategies for managing late blight is to select resistant cultivars using traditional breeding methods that rely on cross-breeding or artificial mutagenesis. However, these methods are time-consuming and laborious, and are limited by the availability of resistant germplasm. Moreover, developing disease-resistant potato cultivars through traditional breeding is particularly challenging because of high heterogeneity and tetraploid inheritance (Muthoni et al., 2015). Therefore, new breeding methods to overcome these hurdles are required.

Genome editing through CRISPR-Cas9 is now a major component of new crop breeding technologies for increasing productivity or disease resistance. In particular, inducing mutations using pre-assembled Cas9 protein-synthesized guideRNA (RNP) transfection into protoplasts is highly advantageous when foreign gene-free plants are required. The lower off-target effects of Cas9 protein-synthesized RNP approaches than that of plasmid-based genome editing is also a positive aspect. Overall, RNP-mediated DNA-free genome editing provides a promising strategy for the rapid commercialization of improved crop cultivars given the stringent policies for handling GMOs. To date, RNP-based CRISPR/Cas9 technology is widely used in maize (*Zea mays*) (Svitashev et al., 2016), bread wheat (*Triticum aestivum*) (Liang et al., 2017), grapevine (*Vitis vinifera*) (Malnoy et al., 2016), apple (*Malus domestica*) (Malnoy et al., 2016), and more recently potato (Andersson et al., 2018; Kieu et al., 2021; Zhao et al., 2021).

In this study, a CRISPR/Cas9 system was used to generate gene edited knock-out potato mutants to the putative S gene, *StSR4*, as a strategy to confer resistance to *P. infestans*. Two guide RNAs (gRNAs) targeting the *StSR4* locus were each introduced into potato protoplasts in association with the Cas9 nuclease protein in the form of pre-assembled RNPs to analyze insertion and deletion (indel) frequencies in regenerated plants. Editing of *StSR4* in potato increased the expression of *StEDS1*, *StPAD4*, and *StPR1*, and triggered an elevation in SA content. Although resistance to late blight improved, increased *StEDS1*, *StPAD4*, and *StPR1* expression coupled with high SA levels had a significant inhibitory effect on plant growth.

## Materials and methods

### Plant materials and Cas9 protein

Potato (*S. tuberosum* cv. Desiree) was used in this study. Plants were grown *in vitro* at a constant temperature of 24°C under 16 h light/8 h dark photoperiod and 80 μE m^−2^ s^−1^ light intensity. Leaves of 2-week-old *in vitro*-grown plants were used as the explant source for protoplast isolation. To conduct protoplast isolation and regeneration, plants were grown in plant containers containing basal Murashige and Skoog (MS; pH 5.8) medium composed of MS salts and vitamins, 3% (w/v) sucrose, and 0.8% (w/v) Plant agar (Duchefa Biochime B.V.). *Streptococcus pyogenes* Cas9 (SpCas9) protein (Thermo Fisher Scientific, Waltham, MA, USA), containing the nuclear localization signal, was used to enhance the rate of genomic DNA cleavage. A CRISPR RNA (crRNA), which contains a specific sequence for guiding the Cas9 protein to the target site in the potato genome, and a trans-acting CRISPR RNA (tracrRNA), which hybridizes to the crRNA to activate the Cas9 enzyme, formed the crRNA: tracrRNA complex.

### Protoplast isolation and purification

Mesophyll protoplasts were isolated from the leaves of *in vitro*-grown potato plants, as described previously (Moon et al., 2021). Briefly, partially wounded leaves (~1 g) were soaked in 30 mL extraction solution, containing 20 mM MES, 1% Cellulase Onozuka R10 (Yakult Pharmaceutical, Inc. Co., Tokyo, Ja Hsu), 0.5% Macerozyme (Yakult Pharmaceutical, Inc. Co., Tokyo, Japan), 0.5 M Mannitol, 20 mM KCl, 10 mM CaCl_2_, and 0.1% BSA (pH 5.7) in the dark at 24°C on a shaker (50 rpm) for 6 h, and then purified using a sucrose cushion to measure their density. The protoplasts were washed twice by resuspending the pellet in 3-fold volume of wash solution (0.24 M NaCl, 2 mg/L NAA, 0.5 mg/L BA in basal medium, pH 5.6) and centrifuged at 50 × *g* for 5 min. Finally, protoplast pellets were resuspended in MMG solution (4 mM MES [pH 5.7], 400 mM mannitol, and 15 mM MgCl_2_) at a final concentration of 1 × 10^6^ protoplasts/mL. To confirm the protoplast density, the number of protoplasts in a 10 µL aliquot of the protoplast suspension was measured using a Fuchs-Rosenthal Hemocytometer chamber under a microscope.

### Target site selection and gRNA design

Three specific gRNAs targeting the *StSR4* gene were designed using the CRISPR RGEN Cas-Designer software (Park et al., 2015). The synthetic crRNAs were purchased from Bioneer Co. (Daejeon, South Korea), and tracrRNA was purchased from Integrated DNA Technologies Co. (Coralville, IA, USA).

### Genotyping

To characterize the *StSR4* exon1 region, the genomic DNA of cv. Desiree was amplified with StSR4_F1(446) and SR4_R-T7 primers (**Table S1**) using 2X *pfu* PCR Master Mix (BioFACT, Daejeon, South Korea). The amplified PCR products were purified and sequenced by Sanger sequencing at Bioneer Co. (Daejeon, South Korea). As a result, a SNP of C/T was observed at the +39bp region of exon1 (**Figure S1**). Therefore, the target site was selected within the sequence except for the site with the SNP.

### RNP assembly

The RNP complex was formed using the Cas9 protein and synthetic crRNA and tracrRNA. First, to generate the gRNA complex, a mixture of crRNA and tracrRNA (1:1 molar ratio) was incubated at room temperature for 10 min. Then, 10 µg each of the Cas9 protein and gRNA complex (1:10 molar ratio) were mixed and incubated at room temperature for 10 min.

### Protoplast transfection

The RNP was introduced into potato protoplasts by polyethylene glycol (PEG)-mediated transformation, as described previously (Nicolia et al., 2015; Woo et al., 2015). Briefly, resuspended protoplasts (3 × 10^5^) were mixed with the pre-assembled RNP and incubated at room temperature for 10 min. Then, an equal volume of freshly prepared 40% (w/v) PEG solution was added and the mixture was incubated at 25°C for 10 min in the dark. After stopping the reaction, the transfected protoplasts were collected by centrifugation at 50 × *g* for 5 min and then gently resuspended in differentiation (D) medium (Nicolia et al., 2015; Moon et al., 2021). Then, an equal volume of alginate solution (2.8% w/v alginic acid-sodium salt and 0.4 M sorbitol) was added to the protoplast suspension, and mixed thoroughly by inverting. After approximately 2 h of incubation in the dark at room temperature, alginate droplets solidified on solid agar (0.4 M sorbitol, 50 mM CaCl_2_·2H_2_O, 2 g/L gelrite, and 6 g/L plant agar) were cultured for plant regeneration.

### T7E1 assay

One week after transfection, targeted mutagenesis in potato protoplasts was detected by the T7E1 assay. Total genomic DNA was isolated from the protoplast population or calli using the HiYield Genomic DNA Mini Kit (Real Biotech Corporation, Taiwan), according to the manufacturer’s instructions. Then, the target *StSR4* site (289 bp) was amplified from the genomic DNA using StSR4_F1 (446) and StSR4_R-T7 primers (**Table S1**), and the PCR product was used for the T7E1 assay as described previously (Kim et al., 2009). Single band PCR products were denatured at 95°C for 5 min and then re-annealed by decreasing the temperature by 1°C per min until reaching 4°C. The hetero-complexed PCR product (15 µL) was incubated with 5 U of the T7E1 enzyme (New England Bio Labs, Ipswich, MA, USA) at 37°C for 30 min. The mismatched products were analyzed by electrophoresis.

### Callus formation and plant regeneration

A method for regenerating plants from micro callus was optimized for our experimental conditions, based on previously reported methods (Moon et al., 2021). Briefly, the transfected protoplasts immobilized in alginate lens were incubated on callus induction medium in the dark at 25°C for 4 weeks, with subculture at weekly intervals, to produce micro callus. Micro calli were transferred to the proliferation medium for mini calli formation, which were subsequently transferred to greening medium. After 6 weeks, greened calli were transferred to regeneration medium and subcultured every 2 weeks with special care to avoid breaking the callus cluster. Regenerated plants from green callus were transplanted into medium and soil by inducing roots. These regenerated plants were obtained by inducing single micro callus and mini callus from protoplast fixed to the alginate lens, and since the callus from which on plant was regenerated was discarded, each regenerated plant can be seen as an individual plant.

### Sanger sequencing assay

PCR-Sanger sequencing analysis can identify mutations or indel efficiencies by sequencing the genomic region surrounding the amplified target site and analyzing the sequence difference from the wild-type sequence through sequence alignment soft (Gaillochet et al., 2021).

To identify CRISPR/Cas9-induced mutations in the regenerated plants, the region flanking the *StSR4* target site was amplified directly from plant tissue with HelixAmpTM Direct PCR [3G] (NANOHELIX, Daejeon, South Korea), according to the manufacturer’s instructions. The 289 bp amplicon was purified using the Expin PCR SV Kit (GeneAll, Seoul, South Korea) and subjected to Sanger sequencing (BioFACT, Daejeon, South Korea). The insertion/deletion mutation (indel) at the target site was analyzed based on the sequencing chromatogram. The frequency of mutagenesis was defined as the number of mutants divided by the total number of regenerated plants (**Figure S2**).

### Next generation sequencing-based targeted deep sequencing and mutation analysis

Genomic DNA was isolated from protoplasts at 3–4 days after transfection and from regenerated potato plants. Libraries were constructed using a two-step PCR protocol. A primary PCR was performed using 2X pfu PCR Master Mix (BioFACT, Daejeon, South Korea) to attach index and sequencing adaptors to both ends of the 289-bp *StSR4* target site containing sequences complementary to gRNA_1 and gRNA_3. To create adaptor overhangs at both ends of the amplicon by primary PCR, the 5’-ILMN preadapter-sequencing primer and sequence-specific locus primer-3’ were designed. Primers SR4_F (adaptor) and SR4_R (adaptor) were purchased from Bioneer. The amplicon was purified using the Expin PCR SV Kit (GeneAll, Seoul, South Korea), according to the manufacturer’s instructions, and was used as the DNA template for the secondary PCR, which was performed using the Nextera XT Index Kit (Illumina, San Diego, CA USA) to attach the index adaptors. Targeted deep sequencing was performed using the Illumina MiSeq platform at Macrogen (Seoul, South Korea). Raw reads for all accessions are available from *stsr4_1* mutants (SRA accession No. SRR20664905-SRR20664984) and *stsr4_3* mutants (SRA accession No. SRR20664783-SRR20664862) in GenBank Sequence Read Archive (SRA) under BioProject number (PRJNA862654).

### Morphological analysis of *stsr4* mutants

The morphology of at least five plants of each *stsr4* mutant line was examined. Single nodes were cut from cultured potato plantlets and cultured in basal MS medium at 24°C, 16 h light/8 h dark photoperiod, and 50% humidity. All plants were photographed daily with a high-resolution camera (Nikon D750 with AF-S Micro Nikkor 105 mm 1:2.8G ED lens; Nikon, Inc., Japan) for up to 30 days. Phenotypic traits including number of branches per plant, number of leaves per branch, stem length, and midrip length were measured using four plants excluding maximum and minimum values from six plants with ImageJ software (https://imagej.nih.gov/ij). Midrip and petiole lengths were measured with an average of three leaves from the first leaf excluding the shoot-tip in each plant. However, the early stage phenotype (0 day-5days) was measured with an average of two leaves in each plant due to the small size plant.

### *P. infestans* resistance assay

To perform the *P. infestans* resistance assay, fully expanded leaves of *stsr4* mutants (selected based on targeted deep sequencing data) and wild-type Desiree plants, grown in a controlled room at 25°C and 16 h light/8 h dark photoperiod, were inoculated with *P. infestans* strain 88069 zoospore suspension (3 × 10^4^ sporangia/mL), as described previously (Ha et al., 2017). The inoculated plants were incubated at 20°C under high humidity (95–100%), and disease symptoms were examined at 5 days after inoculation (DAI). Five days after inoculation, the area of necrotic symptoms to the total leaf area in the whole plant was investigated, and the average ratio (%) of symptom was quantified from high-resolution camera (Nikon D750 with AF-S Micro Nikkor 105 mm 1:2.8G ED lens; Nikon, Inc., Japan) and ImageJ software. All experiments were repeated five times.

### Expression analysis of pathogen related genes

Total RNA was extracted from the leaf tissue using TRIzol Reagent (Invitrogen, Waltham, MA, USA) and then treated with DNase I (Qiagen), according to the manufacturer’s instructions. The total RNA isolated from three biological replicates per treatment was pooled, and the quality of the pooled samples was evaluated using the NanoDrop ND-2000 Spectrophotometer (Thermo Fisher Scientific). Then, first-strand cDNA was synthesized from 1 μg total RNA using the AccuPower^®^ CycleScript™ RT PreMix (Bioneer), according to the manufacturer’s instructions. Quantitative real-time PCR (qRT-PCR) was performed on the CFX Connect Real-Time PCR System (Bio-Rad, Hercules, CA, USA) using the SYBR Green Master Mix (Enzynomics Co., Daejeon, South Korea) and *StEDS1*, *StPAD4*, and *StPR1* specific primers (**Table S1**). The *EF1a* gene was used as an internal control for data normalization. Three biological replicates were performed for each experiment.

### Quantification of SA

Five biological replicates, each containing 10 plants, were performed for each *stsr4* mutant line. The sample used for SA accumulation analysis was harvested from leaves of plants cultured *in vitro* for 20 days in basal MS medium at 24°C, 16 h light/8 h dark photoperiod, and 50% humidity. Leaf tissues were frozen in liquid nitrogen, and then ground with a mortar and pestle. One millilitre of ethylacetate containing 1 µL/mL SA (as an internal standard) was added to approximately 100 mg frozen ground plant tissue in 2 mL EP tubes. The supernatant was evaporated to complete dryness at 30°C for 1 h using a vacuum concentrator. The dried pellet was resuspended in 100 µL of 70% methanol and subjected to liquid chromatography-mass spectrometry (LC-MS) analysis for measuring the SA of the leaves.

### Analysis of candidate off-target editing in mutants

To validate the accuracy of Cas9-RNP-mediated genome editing, eight candidate off-target sites containing 2–4 nt mismatches were identified using the Cas-OFFinder program (http://rgenome.net/cas-offinder/) (Bae et al., 2014). The sequence-specific primer sets (**Table S1**) were designed to PCR amplify the region flanking the eight putative off-target sites from genomic DNA isolated from wild-type and *stsr4* mutants. The amplified PCR products were subjected to NGS-based targeted deep sequencing using the Illumina MiSeq platform (Macrogen, Seoul, South Korea). The raw reads of targeted deep sequencing are available from off-target data (BioSample accession No. SAMN30672769-SAMN30672792) in BioProject number (PRJNA862654).

### Statistical analysis

The statistical significance of the difference between two average values was analyzed using Student’s *t*‐test at *p* < 0.05. Quantitative data are displayed as mean ± standard deviation (SD).

## Results

### Generation of *stsr4* knockout mutants

**Figure 1:**
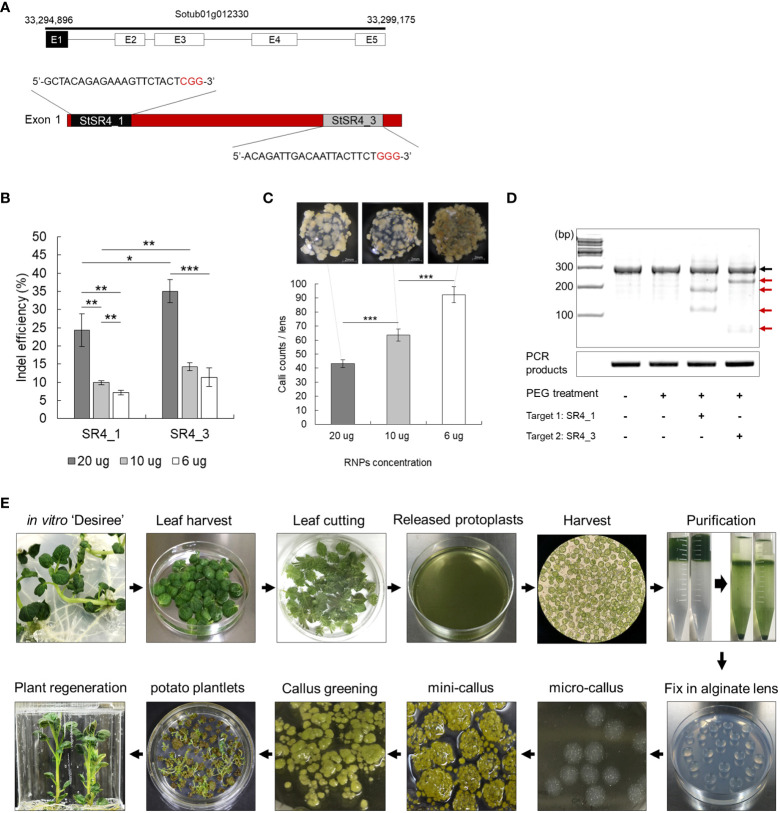
Ribonucleoprotein (RNP)-mediated CRISPR/Cas9 genome editing in potato. **(A)** Identification of target susceptibility (*S*) genes. Selection and design of two crRNA target sites in exon 1 of *StSR4* in the potato cultivar Desiree. Black sequence, crRNA target sequence; red sequence, PAM site. **(B)** Indel efficiency at two target sites, depending on the concentration of the Cas9 protein and guide RNA (gRNA) in protoplasts. Values represent the mean ± SE of three replicated experiments. Student’s t-test; *p < 0.05, **p < 0.01, ***p < 0.001. **(C)** Efficiency of mini callus induction, depending on the concentration of RNPs in protoplasts. Callus growth as a function of RNP concentration: 20 µg, 10 µg, and 6 µg. **(D)** The T7E1 assay of mutations in protoplasts independently treated with SR4_1 and SR4_3 RNPs. Black arrow indicates the wild-type fragment; red arrow indicates indel-carrying fragments. **(E)** Schematic diagram showing the process of inducing plant regeneration from potato protoplast.

Two guide RNAs (SR4_1 and 3) complementary to the exon 1 sequence were designed to knockout the *StSR4* gene (Sun et al., 2016) in the tetraploid potato cultivar ‘Desiree’. The SR4_1 and SR4_3 gRNAs without SNPs in the target sequences were used for subsequent experiments. These targets were confirmed that there appears to be no allelic variation (**Figure 1A**).

**Figure 2:**
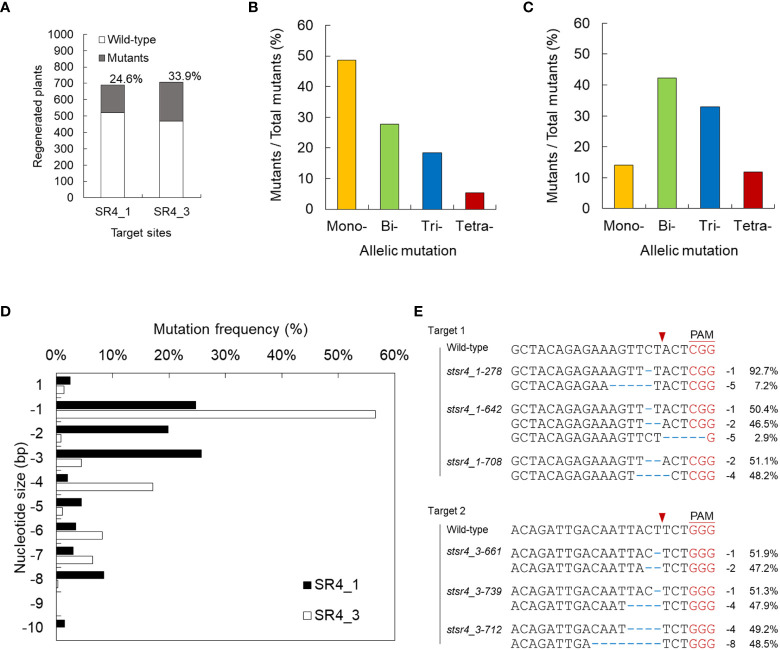
Screening for mutants induced by *StSR4* gene editing of regenerated plants. **(A)** Efficiency of *stsr4* knockout mutant regeneration from protoplasts transfected with SR4_1 and SR4_3 RNPs. A total of 170 and 240 mutants were identified by PCR, followed by Sanger sequencing, among 691 and 708 SR4_1 and SR4_3 transfected protoplast-derived regenerated potato plants, respectively. **(B, C)** Mutation type of mutants induced by the RNPs of SR4_1 **(B)** and SR4_3 **(C)**. Mono-, only one allele is mutated; Bi-, the two alleles are mutated; Tri-, the three alleles are mutated; Tetra-, the four alleles are fully mutated. **(D)** Targeted deep sequencing-based analysis of indel patterns in the regenerated mutants. **(E)** Sequence analysis by targeted deep sequencing of 4 KO *stsr4* mutants.

Overcoming the low editing efficiency of the RNP-mediated CRISPR-Cas9 system remains a challenge. In an attempt to mitigate this problem, the effect of injecting different concentrations of RNP on editing efficiency was examined. Increasing the amount of RNP to 20 µg improved indel efficiency (**Figure 1B**), but simultaneously decreased micro calli formation (**Figure 1C**). The transfection of 20 µg RNP into protoplasts yielded fewer, but larger, light-yellow-colored calli than those of 6 µg RNP, which induced the formation of micro calli to approximately 92.4 calli/6 mm alginate lens mini brown-calli. PEG-mediated transfection of 20 µg RNP into protoplasts resulted in indel frequencies of 24.3 ± 4.5% and 35.1 ± 3.2% for SR4_1 and SR4_3, respectively, as assessed by the T7E1 assay (**Figures 1B, D**). Regenerated plants were successfully induced from RNP-infected protoplasts using the established protoplast-derived regeneration system (**Figures 1E**; **S3**), and 170 and 240 mutants of 24.6% and 33.9% were obtained by Sanger sequencing, respectively (**Figure 2A**; **Table S2**). In addition, mutations were reconfirmed by T7E1 assay in 30 randomly selected mutants (13 *stsr4_1* and 17 *stsr4_3* mutants) (**Figure S2**).

We performed targeted deep sequencing with randomly selected 76 individual mutant lines edited by SR1_1_RNP and 78 by SR4_3. Mutants were obtained in all alleles with frequencies of 3.9% (4 mutants) and 11.8% (9 mutants) in both SR4_1 and SR4_3 target sequences, respectively. Mutations induced in 1, 2, and 3 alleles showed efficiencies of 48.7%, 27.6%, and 18.4% in SR4_1 mutants, respectively, and 14.1%, 42.3%, and 32.9% in SR4_3 mutants, respectively (**Figure 2B**; **Table S2**). In regard to the indel type of each mutant, various deletions of 1, 2, and 3 nt were detected in SR4_1, whereas in the target sequence of SR4_3, 1 nt was deleted with more than 50% frequency, followed by deletion of 4 nt (**Figure 2C**). Finally, six plants carried a frameshift mutation in 90% or more NGS reads obtained from each target site (**Figure 2D**).

### *StSR4* acts as a negative regulator of SA-related gene expression

**Figure 3:**
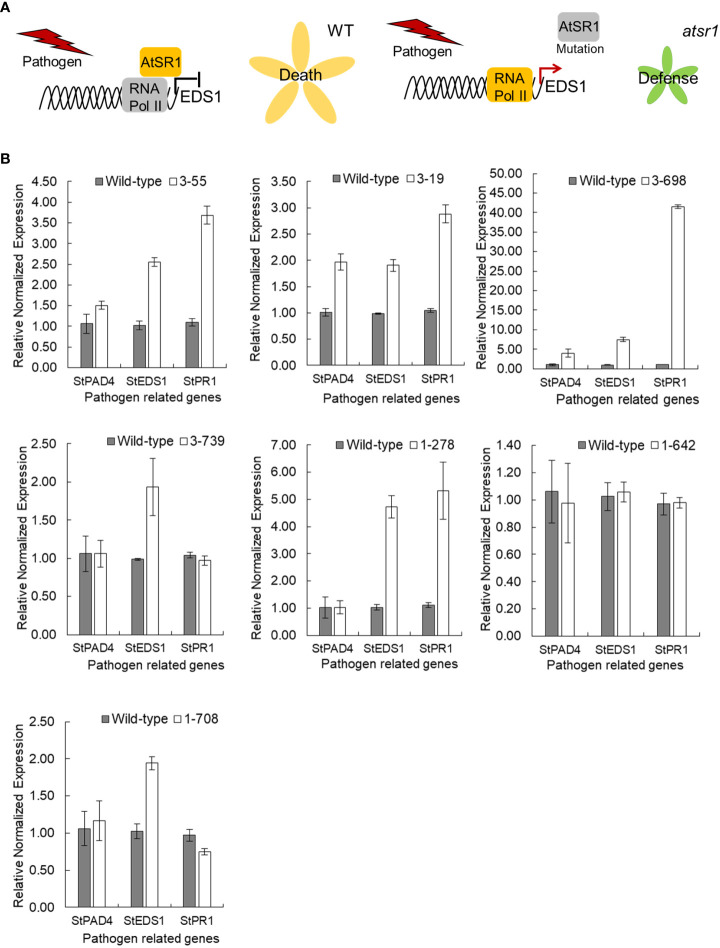
Transcript levels of *StEDS1*, *StPAD4* and *StPR1* in *stsr4* mutants. **(A)** A model demonstrating the function of *AtSR1* as a negative regulator of pathogen resistance. **(B)** Analysis of mRNA expression of SA-related genes, *StEDS1*, *StPAD4*, and *StPR1*, in *stsr4* mutants.

In Arabidopsis, *AtSR1* not only inhibits the expression of several SA immunity pathway genes, but also directly controls the expression of *AtEDS1*, which encodes a putative lipase-like protein that promotes the accumulation of SA through a poorly understood mechanism (Kim et al., 2017) (**Figure 3A**). To determine the effect of *StSR4* gene suppression in potato, transcript levels of the SA pathway-related gene, *StEDS1*, was analyzed by qRT-PCR. *StEDS1* expression was evaluated on randomly selected representative mutants for two allele-types (two allele knockouts, 2KO: *stsr4_3-55*; three allele knockouts, 3KO: *stsr4_3-19* and *stsr4_3-698*), and four allele knockouts (4KO:*srst4_1-278, stsr4_1-642, stsr4_1-708*, and *stsr4_3-739*) (**Figures 2D**; **Figure S4**). On the other hand, single allele knockout (1KO) mutants were excluded from the analysis because it was expected that their growth would not be significantly different from that of wild type. The results showed that relative *StEDS1* transcript levels increased at least 2-fold in the six mutants except for *stsr4_1-642* (**Figure 3B**). In particular, the *stsr4_3-698* mutants showed about 7.51 ± 0.57-fold higher relative *StEDS1* transcript levels. These results indicate that the *StSR4* acts as a negative regulator of *StEDS1* expression.

**Figure 4:**
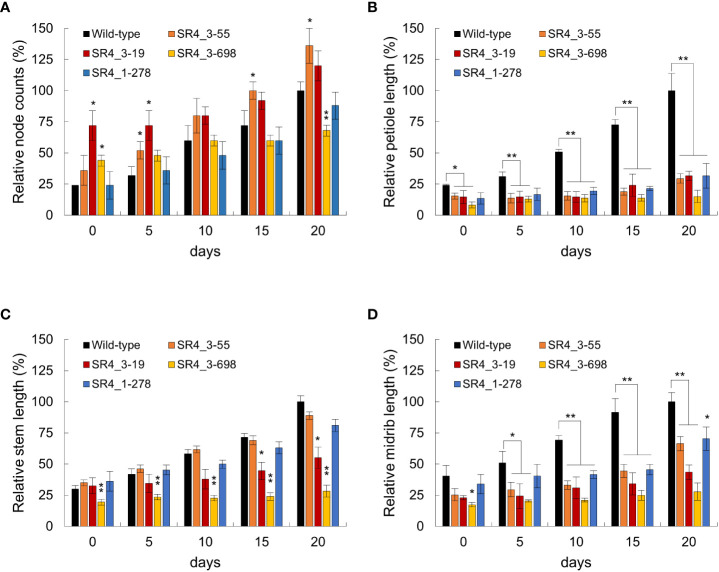
Phenotypic characterization of wild type and *stsr4* mutants (*stsr4_3-55*, *stsr4_3-19, stsr4_3-278*, and *stsr4_3-698*). Plants were grown *in vitro* for 20 days at 16 h light/8 h dark. Representative whole plant phenotypes in wild type and *stsr4* mutants. The typical phenotype with node counts **(A)**, petiole length **(B)**, stem length **(C)**, and midrib length **(D)** of each genotype was observed and photographed every 5 days. Node counts (number), petiole, stem, and midrip length (mm) of 20 days old wild-type plants were 8.3 ± 0.57, 10.73 ± 1.47, 38.37 ± 1.85, and 11.63 ± 0.85, respectively. Values represent the mean ± SE of six replicated experiments (each mean value represents the measured phenotype of four plants). Bars indicate SE. Student’s t-test; *p < 0.05, **p < 0.01.

Because an increase in *EDS1* expression causes a rise in SA levels, the expression of the SA marker gene, *pathogenesis-related gene 1* (PR1), was analyzed in six *StSR4*-edited mutants with elevated *StEDS1* expression (**Figure 3B**). Results showed that relative *StPR1* transcript levels increased in the four mutants, *stsr4_3-19*, *stsr4_3-55*, *stsr4_1-278*, and *stsr4_3-698*. *StPR1* expression increased by 41.55 ± 0.48 fold in the *stsr4_3-698* mutant, which was the highest among the mutants with elevated *StEDS1*. *StPAD4* transcript levels were quantified to determine whether the increased *StPR1* expression is caused by SA accumulation resulting from the interaction between PAD4 and EDS1. Results revealed that relative *StPAD4* expression was significantly higher in *stsr4_3-19* and *stsr4_3-698* mutants, and slightly increased in *stsr4_3-55* mutants. By contrast, *StPAD4* expression did not change in *stsr4_1-278*. Mutating *SR1* in *A. thaliana* leads to a dwarf phenotype because of elevated SA (Du et al., 2009). In addition, the EDS1 and PAD4 protein complex promotes SA biosynthesis (Liang et al., 2017). The *stsr4_3-55* and *stsr4_1-278* mutants with slightly elevated *StPAD4* expression and no change in *StPAD4* transcript levels, respectively, showed a phenotype almost similar to that of wild type except for petiole length (**Figure 4**). The *stsr4_3-19* and *stsr4_3-698* mutants, which showed enhanced expression of *StPAD4* and *StEDS1*, had dwarf phenotypes. The dwarf phenotypes of *stsr4_3-19* and *stsr4_3-698* were expected given that these mutants had elevated SA. The *stsr4_3-19* mutant showed a typical dwarf phenotype, with increased branch numbers and leaves per plant, as well as reduced midrib and stem lengths (**Figure 4**). The *stsr4_3-698* mutant, which had the highest levels of *StPR1* transcripts among the mutants, displayed inhibited stem and leaf growth, and had almost no growth for 20 days *in vitro*. Because of the severely inhibited growth phenotype of *stsr4_3-698*, it was difficult to obtain samples to further evaluate this mutant. As such, subsequent experiments excluded analysis of the *stsr4_3-698* mutant.

### High SA content in *stsr4* mutants leads to enhanced pathogen resistance

**Figure 5:**
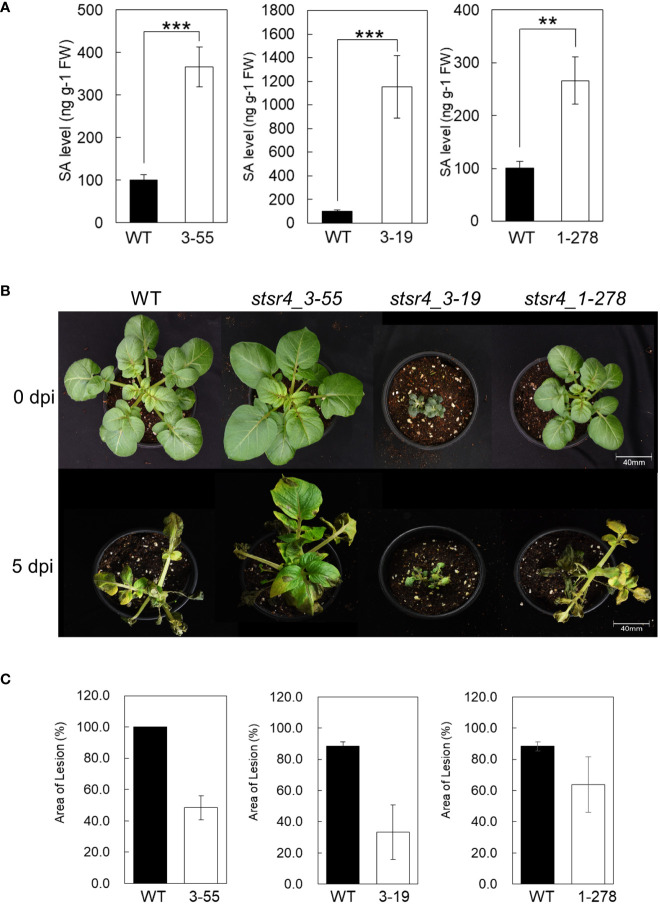
Plant immune response through the suppression of *StSR4* expression is regulated by SA. **(A)** Quantification of free SA in three *stsr4* mutants (*stsr4_3-55, stsr4_3-19*, and *stsr4_1-278*). Data represent the mean ± SE of five biological replicates. Asterisks indicate significant differences (Student’s *t*-test; **p < 0.01, ***p < 0.001). **(B)** Growth at 25°C for 3 weeks in pots of three mutants before pathogen resistance experiment. **(C)** Disease symptoms observed on the leaves of wild type and *stsr4_3-55* and *stsr4_3-19* mutant plants at 0 days post-inoculation (dpi) and 5 dpi with *Phytophthora infestans*. Leaves of wild type and *stsr4* mutant plants grown at 25°C for 3 weeks were sprayed with a zoospore suspension of *P. infestans* strain 88069. At least three biological replicates were performed.

Results thus far showed that *StSR4* editing triggered increased *StEDS1* and *StPR1* expression. Endogenous SA content in three *stsr4* mutants, namely, *stsr4_3-19*, *stsr4_3-55*, and *stsr4_1-278*, was measured to determine whether increased *StPR1* expression and the dwarf phenotypes were caused by elevated SA through interactions with *StPAD4.* The *stsr4_3-19* mutant, which had increased expression of *StPAD4* and *StEDS1*, accumulated the highest amount of SA of 1,153 ng/g FW. This increase in SA level in *stsr4_3-19* was significantly higher than that of the wild type by 11.45-fold. The *stsr4_3-55* mutant had SA levels of 366 ng/g FW, while the *stsr4_1-278* mutant, which had the lowest *StPAD4* expression, showed an SA content of 266 ng/g FW. Endogenous SA content in *stsr4*_1-278 was 2.6-fold higher than that of wild type (**Figure 5A**). It is worth noting that *stsr4*_3-19, which had higher SA levels than those of *stsr4_3-55* and *stsr4_1-278*, also had the most severe dwarf phenotype among the mutants analyzed (**Figures 5A, B**).

High SA accumulation enhances the plant innate immunity system, and therefore improves plant resistance to pathogens (Ryals et al., 1996). *P. infestans*, which is the causal organism of potato late blight disease, was inoculated into leaves of three *stsr4* mutants to determine whether increased SA content affects resistance to pathogens. Results revealed that at 5 DAI, most tissues of wild-type were necrotic. By contrast, tissues of *stsr4* mutants had fewer necrotic lesions than those in wild type. For example, the leaf area of the *stsr4_3-55* mutant was only 48.3% necrotic. Furthermore, leaf area of the *stsr4_3-19* mutant, which had the highest SA content, was only 38% necrotic, and therefore exhibited the highest resistance to *P. infestans* among the *stsr4* mutants (**Figure 5C**). However, *stsr4_1-278* mutant, which showed the lowest SA levels, had weak resistance with 72% of its leaf area exhibiting necrosis. These results suggest that SA accumulation in potato improves resistance to pathogens, and that increased *StEDS1* expression resulting from *StSR4* editing increases SA content through its interaction with *StPAD4*.

**Figure 6:**
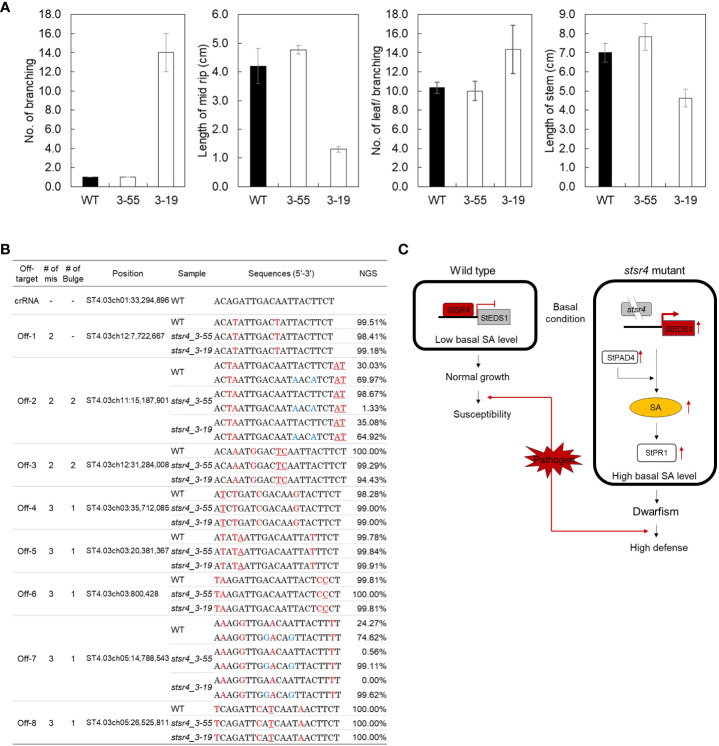
*In vivo* phenotype assays and off-target analysis of two mutants showing improved pathogen resistance. **(A)** Measurement of branch number per plant, midrib length, leaf number per branch, and stem length in wild type and *stsr4_3-55* and *stsr4_3-19* mutants. The plants were grown in a greenhouse under 16 h light/8 h dark photoperiod for 1 month. Values represent mean ± SE of four biological replicates. Asterisks indicate significant differences (Student’s *t*-test; *p < 0.05). **(B)** Analysis of off-target editing in two *stsr4* mutants. Eight candidate off-target sequences were analyzed by PCR, followed by targeted deep sequencing (Read counts > 100). The mismatched nucleotides between the on- and off-target sequences are indicated in red, and the bulges are underlined red. Blue sequences represent SNPs. **(C)** Model explaining the basis of increased SA levels and pathogen resistance in potato, based on the suppression of *StSR4* expression, and consequently increased expression of *StEDS1 and StPAD4*.

As noted earlier, the *stsr4_3-55* mutant had a wild-type phenotype, whereas the *stsr4_3-19* mutant exhibited a dwarf phenotype when grown on MS medium. Two *stsr4* mutants were grown for 1 month in soil to determine whether the *stsr4_3-19* dwarf phenotype was still observed under a different growth environment (**Figure 6A**). Similar to results obtained from MS medium, the *stsr4_3-55* mutant showed a wild-type phenotype, whereas the dwarf phenotype was maintained in the *stsr4_3-19* mutant. These results suggest that the dwarf phenotype of *stsr4_3-19* mutant was caused by *StSR4*–triggered SA accumulation, rather than an environmental effect. The results with potato described here are consistent with a previous report showing that inhibition of SR1 induces a dwarf phenotype in *A. thaliana* (Du et al., 2009).

### Off-target editing in two *stsr4* mutants

The CRISPR-Cas9 technology has the potential to cause off-target mutations at sites similar to the target genomic sequences (Peng et al., 2016). To determine whether genome editing using RNPs can cause off-target editing, we validated mutations in eight candidate off-target sites including 2–4 mismatches with 1–2 bulges in *stsr4_3_55* and *stsr4_3_19* mutants (**Figure 6B**). No indels were observed at the eight candidate off-target sites in the two mutants, based on the analysis of Sanger sequencing-generated chromatograms and Targeted deep sequencing.

## Discussion

CRISPR/Cas9-mediated genome editing is a promising approach for enhancing specific agricultural traits of plants, while preserving their existing elite characteristics through targeted gene mutation. To improve pathogen resistance of the potato cultivar ‘Desiree’, an *S* gene (*SR4*) was edited in potato protoplasts using RNP-mediated genome editing. A first step was to improve gene editing efficiency by considering RNP concentration. Optimizing the concentration of gRNA and Cas9, which are components of the RNP, is important for gene editing. According to previous studies, increasing the molar concentration of gRNA, rather than using the 1:1 molar ratio of Cas9:gRNA, increases the indel efficiency (Min et al., 2019; Wei et al., 2020). The editing efficiency of RNP-mediated genome editing using the 1:10 molar ratio of Cas9:gRNA has been reported, but not applied, in plants (Min et al., 2019). Accordingly, we compared the editing efficiency obtained using different concentrations of RNPs, based on the 1:10 molar ratio of Cas9:gRNA. At high RNP concentrations, the editing efficiency was improved but the micro calli induction rate was decreased, whereas low RNP concentrations decreased the editing efficiency and increased the micro calli induction rate (**Figure 1C**). Excessive induction of callus within a confined space of the alginate lens hinders callus growth (Moon et al., 2021). This indicates that callus size is an important factor affecting plant regeneration. Therefore, 20 µg RNPs, which showed the highest mutation efficiency and lowest callus induction rate, was determined to be an appropriate concentration for genome editing in this study. This implies that mutation efficiency is more important than callus induction rate.

The gRNA could be used as a pre-assembled RNP complex of synthetic crRNA and tracrRNA or as an *in vitro* transcribed RNA (IVT-RNP) derived from a DNA template. Synthetic gRNA might provide possible benefits of increasing the specificity of gRNA, though IVT-RNP can lead to the insertion of the residual DNA template (Andersson et al., 2018). Here, using a high-efficiency protoplast regeneration system as described (Moon et al., 2021) along with two different StSR4-specific gRNAs, low mutation frequency of RNPs, the higher mutation frequency of 25% (170 mutants from 691 regenerated plants) and 34% (240 mutants from 708 regenerated plants) stsr4 mutants were obtained. These results show that the indel efficiency observed in protoplasts extend to regenerated plants. In summary, in order to improve the efficiency of DNA-free genome editing, a high-efficiency protoplast-derived plant regeneration system, appropriate RNP infection concentration, and gRNA target site selection should be considered as factors. In addition, it is necessary for stable mutant supply to ensure that protoplast-level mutation efficiency can be maintained until the regeneration process (**Figure S3**).

Furthermore, we investigated the reason for the difference in indel efficiency at the two target sites of *StSR4*. Although Cas9 activity is affected by the sequence specificity of gRNA (Doench et al., 2014), there was no difference within the sequence of the *StSR4* two target sites; therefore, indel efficiency at the two target sites is unlikely to be affected by Cas9 activity. Rather, the existence of mononucleotide (T) repeats at +1 and +4 bp, where frequent deletions were observed in the SR4_3 target sequence (**Figure 2C**), would have been more effective in creating a frame shift and is considered to have a higher mutation rate than SR4_1 (**Figure 2B**). As such, the targeting specificity of Cas9 is tightly controlled by the 20 nt sequence of gRNA and the presence of PAM, but potential off-target effects can still occur in the genome (Zhang et al., 2015). On the other hand, the RNP-mediated CRISPR/Cas9 system cleaves the target DNA site and is rapidly degraded by endogenous proteases, thereby limiting off-target effects (Foster et al., 2018; Wang et al., 2018). Additionally, we found no evidence of off-target mutations at the eight off-target candidate sites in 16 mutants, supporting the finding that RNP-mediated genome editing is highly specific in plants.

Suppression of the *S* gene, a negative regulator of plant defense, improves resistance against various diseases (Pessina, 2016; Kieu et al., 2021). In this study, improved late blight disease resistance was expected through the suppression of the *S* gene, *StSR4*, in potato. The *stsr4* mutants exhibited improved resistance to *P. infestans* (**Figure 5C**). *StSR4* editing in potato triggered increased expression of *StEDS1*, which led to higher transcript levels of the pathogen resistance marker gene, *StPR1* (**Figure 3B**). These results are consistent with reports of increased SA accumulation and pathogen resistance following the suppression of *SR1* expression in Arabidopsis (Du et al., 2009). However, SA levels did not increase in all *stsr4* mutants with high *StPR1* expression. SA accumulation in the three mutants with high *StPR1* expression was proportional to *StPAD4* transcript levels. This observation suggests that *StPAD4* expression is related to *StEDS1*-induced SA accumulation. These results support a previous report showing that EDS1 and PAD4 play a parallel role in SA synthesis in *A. thaliana* basal- and effector-triggered immunity (Liang et al., 2017).

An increase in SA accumulation provides improved immunity to plants but also inhibits plant growth, as supported by the suppression of plant growth because of SA accumulation (Galon et al., 2008; Du et al., 2009; Nie et al., 2012). In this study, the *stsr4_3-19* mutant, in which *StSR4* was edited in three alleles by CRISPR/Cas9, showed a significantly high level of SA, and improved resistance to *P. infestans*. However, growth of this mutant was also severely inhibited as manifested by a dwarf phenotype (**Figure 4**). Furthermore, the *stsr4_3-698* mutant with *StSR4* edited in three alleles did not survive because growth was strongly inhibited along with significantly high *StEDS1*, *StPAD4*, and *StPR1* transcript levels, which were associated with high SA. By contrast, the *stsr4_3-55* mutant with *StSR4* edited in two alleles, showed a slight increase in SA and a wild-type phenotype, except for short petioles. Therefore, high SA content triggered by *StSR4* editing in potato not only improves resistance to pathogens, but also induces a dwarf phenotype. Here, we did not obtain mutants with improved *P. infestans* resistance in the full allele mutants. In the *stsr4_1-278* mutant, the expression of *StEDS1* and *StPR1* increased (**Figure 3B**), which indicated that *StSR4* was strongly suppressed by the allele mutation. Nevertheless, the *stsr4_1-278* mutant showed low *StPAD4* expression and SA levels, which resulted in weaker resistance to *P. infestans* than that in *stsr4_3-55* or *stsr4_3-19* mutants. In addition, with low SA content, the plant recovered from dwarf, unlike the *stsr4_3-19* mutant, and was phenotypically similar to the wild type. Previously, in the *atsr1* mutant, overexpression of the *NahG* gene, which encodes a SA degrading enzyme, reduced SA accumulation and restored the wild-type phenotype (Du et al., 2009). However, *NahG* overexpression led to the suppression of *AtPR1* expression, unlike the results of the current study, which showed high *StPR1* expression in the *stsr4_1-278* mutant. Next, we investigated whether the function of *StSR4* in the *stsr4* mutants could be complemented by its functionally redundant homologs. It has been previously reported that the loss of function of the *eIF4E1* gene by CRISPR/Cas9-based editing is compensated by *eIF4E2*, a homolog of *eIF4E1* (Bastet et al., 2017). We analyzed the expression of *StSR4* gene homologs (accession no.: XM_006337904, XM_006352111, XM_006355330, and XM_006349770) to investigate the effect of gene redundancy in the *stsr4_1-278* mutant; however, we could not confirm any association between the knockout mutation of *StSR4* and the expression of *StSR4* gene homologs (**Figure S5**). Because the accumulation of SA is regulated by various factors, such as the phenylalanine ammonia-lyase pathway and isochorismate pathway, the recovery of SA expression in the obtained full allele mutants is expected to be influenced by factors other than SA degrading enzymes or gene redundancy. In summary, the *SR4* full allele mutation induces a greater increase in SA, which strongly inhibits plant growth, making it difficult to obtain a full knockout mutant, which is considered to be the cause of low regeneration efficiency. However, in some of the obtained full knockout mutants, the expression of SA-related genes was suppressed due to unknown factors, resulting in lower resistance to *P. infestans* and recovering normal plant growth (**Figure S6**).

In conclusion, the genome editing strategy utilized in this study enables the development of disease-resistant genotypes by editing the *S* gene *via* the delivery of synthetic RNPs. We show that RNP concentration affects gene editing efficiency and plant regeneration (**Figure 1E**). This indicates the importance of selecting an appropriate RNP concentration for the alginate method. Both targets of the *StSR4* gene were efficiently edited, and plant regeneration was successfully induced, without off-target mutations in potato protoplasts. Increased expression of *StEDS1* and *StPAD4* in *stsr4* mutants induced SA accumulation, and improved resistance to *P. infestans* (**Figure 6C**). This implies that the function of *StSR4* in potato is similar to that of *AtSR1* in *A. thaliana*. For the first time to our knowledge, we developed *P. infestans*-resistant potato through RNP-mediated genome editing method of the susceptibility gene ‘*StSR4*’ and investigated the mechanism by which the resistance works. This is expected to contribute to encouraging the development of foreign gene-free potato cultivars through genome editing in the future.

## Data availability statement

The original contributions presented in the study are included in the article/**Supplementary Material**. Further inquiries can be directed to the corresponding authors.

## Author contributions

H-SK and Y-iP, conceptualized and supervised the study; K-BM performed the experiments, analyzed the results, and wrote the first draft of the manuscript; S-JP, J-SP, SS, and J-HJ performed experiments involving protoplast isolation, callus induction, shoot regeneration, and plant cultivation; SL and GC performed *P. infestans* inoculation experiments; S-GK measured the SA content; HC, Y-SK, and H-JL analyzed the data and revised the manuscript. All authors contributed to the article and approved the submitted version.
