# Corrigendum: Editing of *StSR4* by Cas9-RNPs confers resistance to *Phytophthora infestans* in potato

**DOI:** 10.3389/fpls.2022.1063362

**Published:** 2023-05-24

**Authors:** Ki-Beom Moon, Su-Jin Park, Ji-Sun Park, Hyo-Jun Lee, Seung Young Shin, Soo Min Lee, Gyung Ja Choi, Sang-Gyu Kim, Hye Sun Cho, Jae-Heung Jeon, Yong-Sam Kim, Youn-Il Park, Hyun-Soon Kim

**Affiliations:** ^1^ Plant Systems Engineering Research Center, Korea Research Institute of Bioscience and Biotechnology, Daejeon, Republic of Korea; ^2^ Department of Biosystems and Bioengineering, KRIBB School of Biotechnology, University of Science and Technology, Daejeon, Republic of Korea; ^3^ Department of Functional Genomics, KRIBB School of Bioscience, University of Science and Technology, Daejeon, Republic of Korea; ^4^ Center for Eco-Friendly New Materials, Korea Research Institute of Chemical Technology, Daejeon, Republic of Korea; ^5^ Department of Biological Sciences, Korea Advanced Institute for Science and Technology, Daejeon, Republic of Korea; ^6^ Genome Editing Research Center, Korea Research Institute of Bioscience and Biotechnology, Daejeon, Republic of Korea; ^7^ GenKOre, Daejeon, Republic of Korea; ^8^ Department of Biological Sciences, Chungnam National University, Daejeon, Republic of Korea

**Keywords:** RNPs, protoplast, genome editing, susceptibility gene, late blight

**Text Correction - metadata**

In the published article, there was an error in affiliation 4. This sentence previously stated “Department of Biological Sciences, KAIST, Daejeon, South Korea”

it should be “Center for Eco-Friendly New Materials, Korea Research Institute of Chemical Technology, Daejeon, Republic of Korea”.

The authors apologize for this error and state that this does not change the scientific conclusions of the article in any way. The original article has been updated.

