# Supplementary material for: Editing of StSR4 by Cas9-RNPs confers resistance to Phytophthora infestans in potato
Source: Front Plant Sci. 2022 Sep 23;13:997888. doi: 10.3389/fpls.2022.997888 (PMC9539116; doi:10.3389/fpls.2022.997888)
Supplement: Supplementary file 1 [file DataSheet_1.pdf]

Table S1. Primers used in this study.

Gene	Primer name	Sequence (5' - 3')	Product size
StSR4	StSR4_F1(446)	AGT TCT CGA GGT CGC AGG AG	278
	SR4_R-T7	GAG GGA CAC CAA TGA CTA CAT G	
StSR4	SR4_F (adapter)	TCG TCG GCA GCG TCA GAT GTG TAT AAG AGA CAG AGT TCT CGA GGT CGC AG	356
	SR4_R (adapter)	GTC TCG TGG GCT CGG AGA TGT GTA TAA GAG ACA GGA GGG ACA CCA ATG AC	
StEDS1	StEDS1_F(q)	GAC ACA GTT TCG CAG GCA AG	265
	StEDS1_R(q)	CCA GCC ATT TGC AGC TGT TT	
StPAD4	StPAD4_F	GAC TCA GCT TTT CCG CGT TG	
	StPAD4_R	TGC TTC ATA GCT CGA CTC CG	
StPR1	StPR1_F(q)	CAG CTG TGC AAT TGT GGG TG	201
	StPR1_R(q)	GTT GTC CGA CCC AGT TTC CA	
StEF1a	EF1a_F(q)	CTT GAC GCT CTT GAC CAG ATT	61
	EF1a_R(q)	GAA GAC GGA GGG GTT TGT CT	
StCAMTA2	StCAMTA2_F	AGG AAG TTC ATC ACC GCT GG	226
	StCAMTA2_R	CTG CCT GCC GTG AAC TTT TC	
StCAMTA4	StCAMTA4_F	TGG ATC CAA CCC TGA CGA AT	202
	StCAMTA4_R	CCA CGT GCA CTC TGA AGG AT	
StCAMTA5	StCAMTA5_F	TGG TGA TTT GCA GGA TGG CT	200
	StCAMTA5_R	AAT CAC CTG CAC CCA CTG TC	
StCAMTA6	StCAMTA6_F	GTC CTG TCT TCT ACT GAT TGA CA	231
	StCAMTA6_R	AGG AGA CAC CAT TGA GGC TG	

Table S2 . Summary of screening in *stsr4* mutants

	Target sequence (5’-3’)	No. of regenerated plants	No. of mutants	No. of mutants for screening by NGS	No. of full allele mutation	No. of full allele KO
StSR4_1	GCTACAGAGAAAGTTCTACT	691	170 (24.6%)	76	4 (4%)	3
StSR4_3	ACAGATTGACAATTACTTCT	708	240 (33.9%)	78	9 (12%)	3

Table S3. Specific primer sets for 8 candidate off-target sites

Off-target	Primers	Sequence (5'->3')	Length (bp)	Product length (bp)
OT1	Forward	CAAAAACCTTGCCTACAAATGCAC	24	290
	Reverse	CGTCGTTTGGGATCGGAGAAT	21	
OT2	Forward	CCATCTGTTAGATTATGTGGCATT	24	282
	Reverse	ACCACCACTATTTATAGGTGAGGAA	25	
OT3	Forward	CATGACAAATTCATTATTTATCCAA	25	297
	Reverse	GTGAAGATCGAGTTAGCAAAAA	22	
OT4	Forward	TTGGACAAAGCTTGGTTCTTT	21	280
	Reverse	TATATGTTAGTTTTCTTCCTTCTCA	25	
OT5	Forward	ACAACCTCTTGCAAAAATGC	20	280
	Reverse	TGAATCTACTTTGATTTCTCCTTGT	25	
OT6	Forward	TCATTATTTATCCAATGACAAGTAT	25	285
	Reverse	GAAGATCGAGTTAGCAAAAATGA	23	
OT7	Forward	AAAATAATCAATGTGTTGTGAACTC	25	250
	Reverse	TTCAGAATCATACTTAACTGCCT	23	
OT8	Forward	TTGACTTGGTTGAAAGGGGA	20	280
	Reverse	GTAAAATTATCTTGTGCACTAACTG	25	
