# Supplementary material for: Editing of StSR4 by Cas9-RNPs confers resistance to Phytophthora infestans in potato
Source: Front Plant Sci. 2022 Sep 23;13:997888. doi: 10.3389/fpls.2022.997888 (PMC9539116; doi:10.3389/fpls.2022.997888)
Supplement: Supplementary file 2 [file DataSheet_2.pdf]

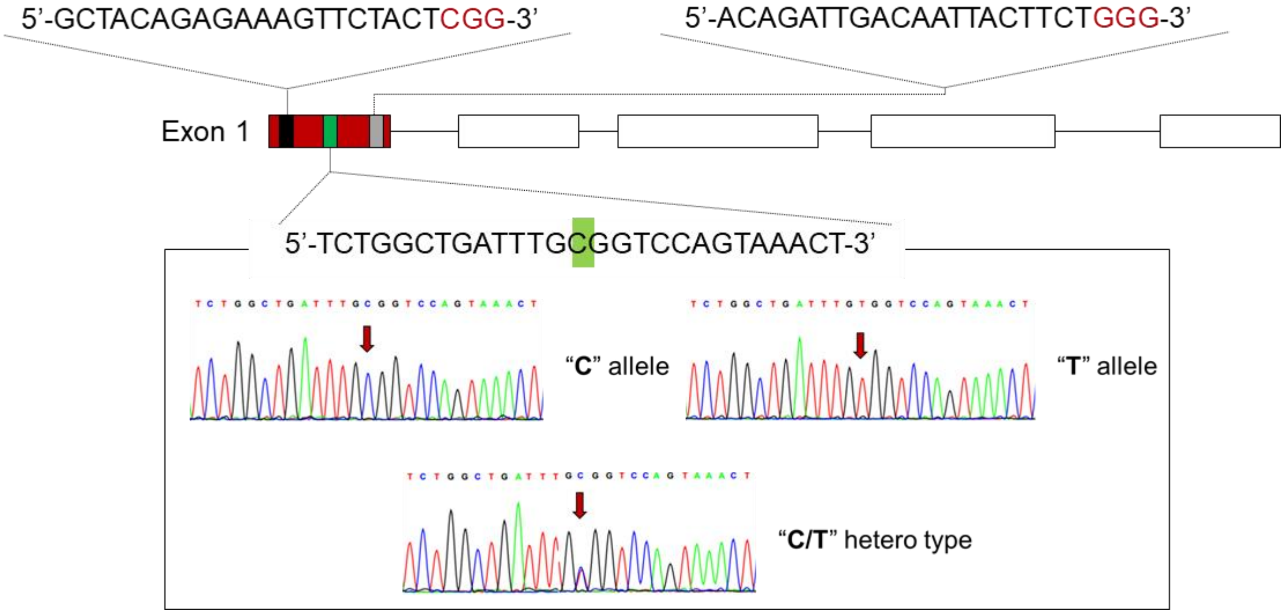

Figure S1. Genotyping of *StSR4* target site for gRNA design. The "C/T" hetero type in exon 1 of *StSR4* gene was confirmed by PCR-Sanger sequencing

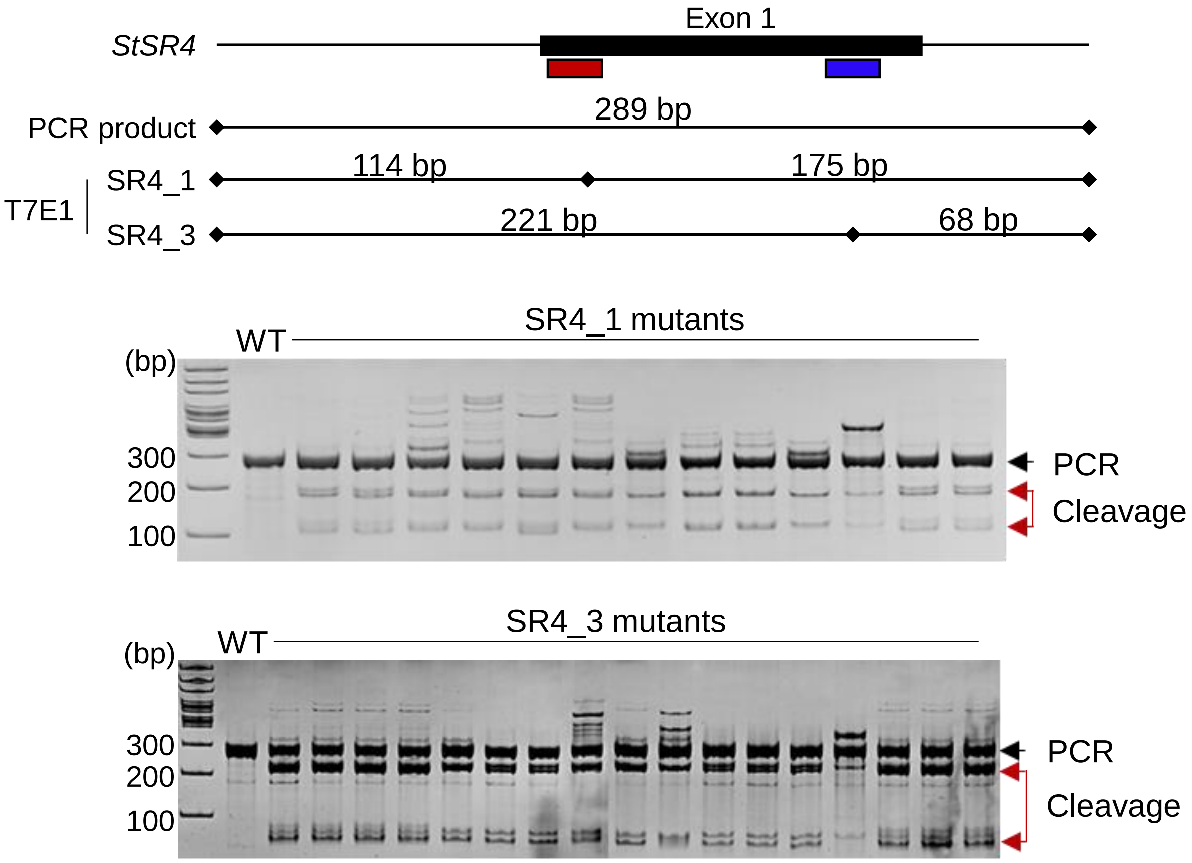

Figure S2. T7E1 assay of mutations in *stsr4_1* and *stsr4_3*. The T7E1 assay was performed to detect mutations in the *StSR4* gene from protoplast-derived regenerated plants using SR4_1 RNPs and SR4_3 RNPs. Black arrow indicates the wild-type fragment; red arrow indicates indel-induced fragments.

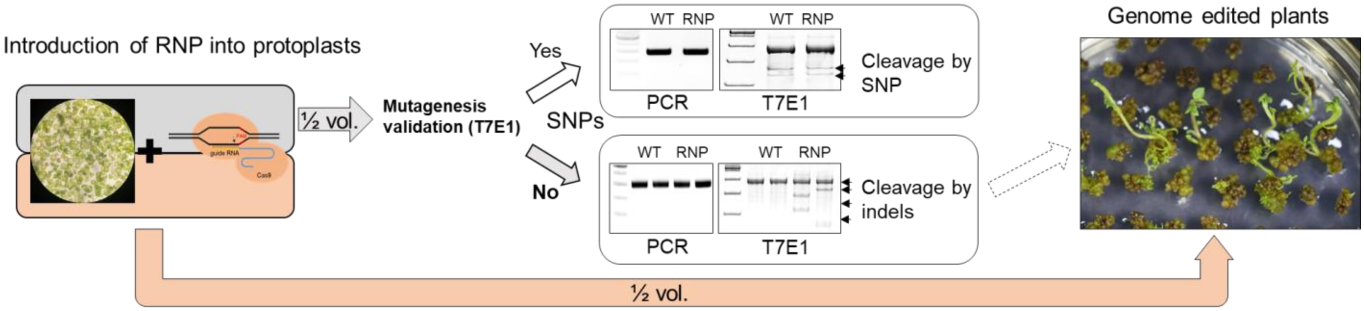

Figure S3. Schematic diagram of a method for efficiently obtaining RNP-mediated gene edited plants. A stable gene edited mutants were efficiently obtained by establishing a system for simultaneously performing indel analysis and culturing of RNP-transfected protoplasts

stsr4_3-55	GCGAACAACAGAAACAGATTGACAATTACTTCTGGGGAAG	WT
	GCGAACAACAGAAACAGATTGACAATTACTTCTGGGGAAG	WT
	GCGAACAACAGAAACAGATTGACAATTAC-TCTGGGGAAG	-1
	GCGAACAACAGAAACAGATTGACAAT----TCTGGGGAAG	-4
stsr4_3-19	GCGAACAACAGAAACAGATTGACAATTACTTCTGGGGAAG	WT
	GCGAACAACAGAAACAGATTGACAATTAC-TCTGGGGAAG	-1
	GCGAACAACAGAAACAGATTGACAAT----TCTGGGGAAG	-4
	GCGAACAACAGAAACAGATTGAC-------TCTGGGGAAG	-7
stsr4_3-698	GCGAACAACAGAAACAGATTGACAATTACTTCTGGGGAAG	WT
	GCGAACAACAGAAACAGATTGACAATTAC-TCTGGGGAAG	-1
	GCGAACAACAGAAACAGATTGACAATTAC-TCTGGGGAAG	-1
	GCGAACAACAGAAACAGATTGACAAT----TCTGGGGAAG	-4

Figure S4. Sequencing of *stsr4_3-55* (2 KO), *stsr4_3-19* (3 KO), and *stsr4_3-698* (3 KO) mutants by targeted deep sequencing. The blue sequence indicates the gRNA target site. The PAM regions are shown in red. The minus (-) symbols indicate the number of nucleotides deleted in the target site.

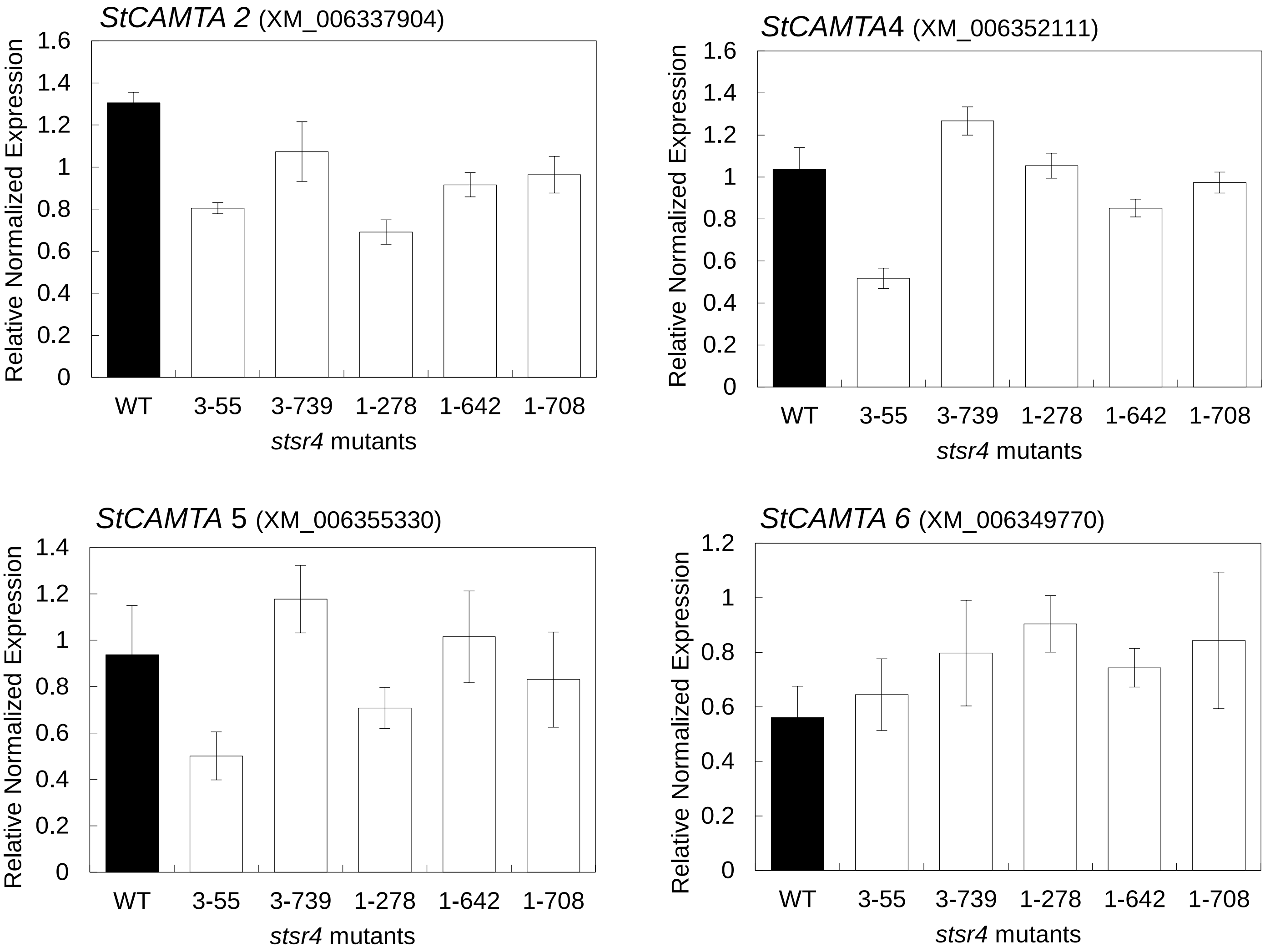

Figure S5. Expression of *StSR4* homologous genes in the 4 KO *stsr4* mutants. Transcript levels of *StSR4* homologous genes (accession numbers: XM_006337904, XM_006352111, XM_006355330, and XM_006349770) in *stsr4* full knockout mutants. A disease-resistant mutant line, *stsr4_3-55*, was used as a control group. Data represent the mean ± SE of three biological replicates.

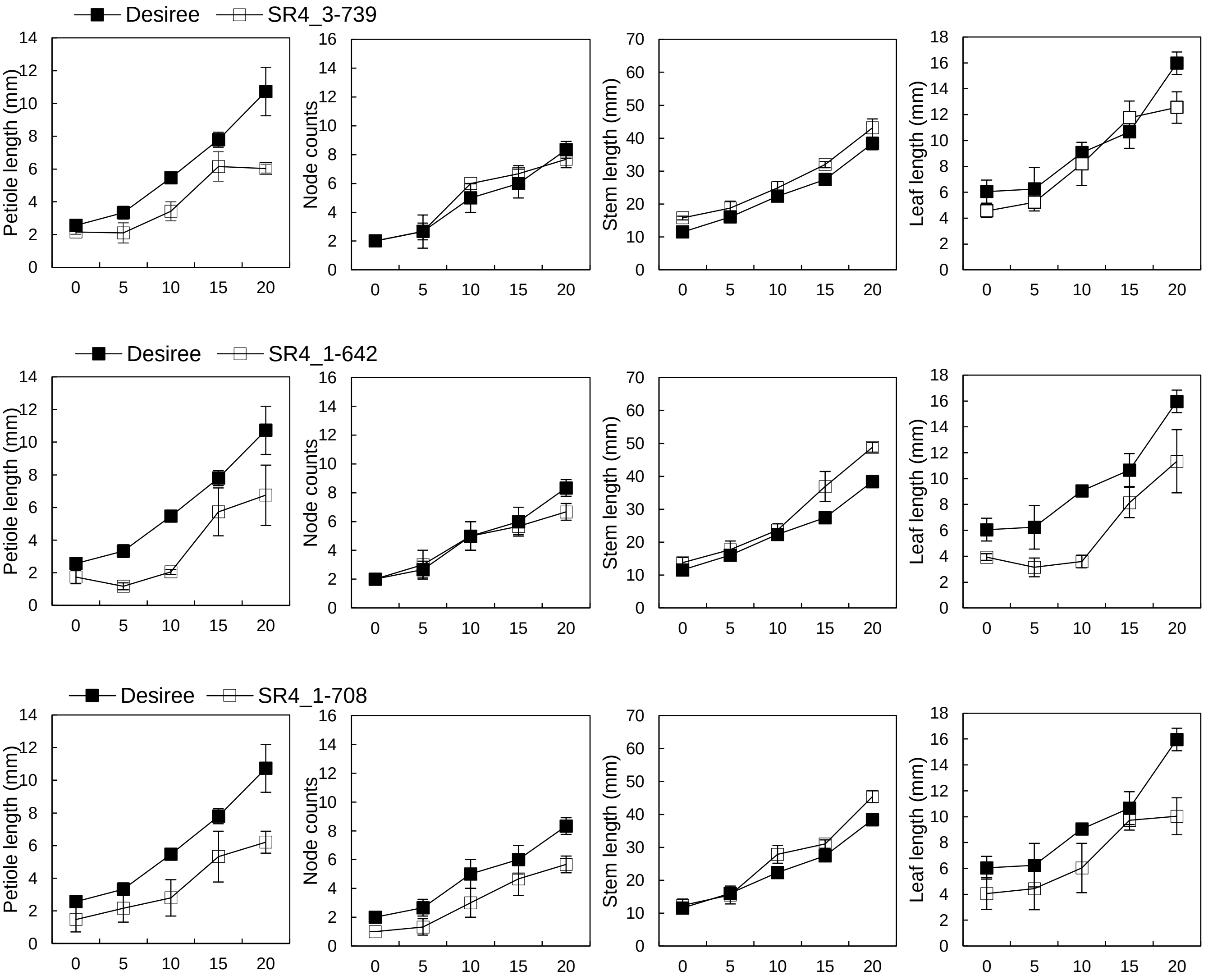

Figure S6. Phenotypic characterization of 3 mutants (*stsr4_3-739*, *stsr4_1-642*, *stsr4_1-708*). Representative whole-plant phenotypes in wild type (cv. Desiree) and *stsr4* mutants. The typical phenotype (with petiole length, node counts, stem length, and leaf length) of each genotype was observed and photographed every five days. Values represent the mean ± SE of six replicated experiments (each mean value represents the measured phenotype of four plants).
